# Two Portable Recombination Enhancers Direct Donor Choice in Fission Yeast Heterochromatin

**DOI:** 10.1371/journal.pgen.1003762

**Published:** 2013-10-24

**Authors:** Tadas Jakočiūnas, Lærke Rebekka Holm, Janne Verhein-Hansen, Ala Trusina, Geneviève Thon

**Affiliations:** Department of Biology, University of Copenhagen, BioCenter, Copenhagen, Denmark; Brandeis University, United States of America

## Abstract

Mating-type switching in fission yeast results from gene conversions of the active *mat1* locus by heterochromatic donors. *mat1* is preferentially converted by *mat2-P* in M cells and by *mat3-M* in P cells. Here, we report that donor choice is governed by two portable recombination enhancers capable of promoting use of their adjacent cassette even when they are transposed to an ectopic location within the *mat2-mat3* heterochromatic domain. Cells whose silent cassettes are swapped to *mat2-M mat3-P* switch mating-type poorly due to a defect in directionality but cells whose recombination enhancers were transposed together with the cassette contents switched like wild type. Trans-acting mutations that impair directionality affected the wild-type and swapped cassettes in identical ways when the recombination enhancers were transposed together with their cognate cassette, showing essential regulatory steps occur through the recombination enhancers. Our observations lead to a model where heterochromatin biases competitions between the two recombination enhancers to achieve directionality.

## Author Summary

The state of chromatin, heterochromatin or euchromatin, affects homologous recombination in eukaryotes. We study mating-type switching in fission yeast to learn how recombination is regulated in heterochromatin. Fission yeast exists as two mating-types, P or M, determined by the allele present at the expressed *mat1* locus. Genetic information for the P and M mating-types is stored in two silent heterochromatic cassettes, *mat2-P* and *mat3-M*. Cells can switch mating-type by a replication-coupled recombination event where one of the silent cassettes is used as donor to convert *mat1*. Mating-type switching occurs in a directional manner where *mat2-P* is a preferred donor in M cells and *mat3-M* is preferred in P cells. In this study, we investigated factors responsible for these directed recombination events. We found that two portable recombination enhancers within the heterochromatic region compete with each other and direct recombination in a cell-type specific manner. We also found that heterochromatin plays an important role in directionality by biasing competitions between the two enhancers. Our findings suggest a new model for directed recombination in a heterochromatic domain and open the field for further studies of recombination regulation in other chromatin contexts.

## Introduction

Fission yeast cells switch mating type by directed recombination events where the information in the expressed *mat1* locus is replaced with information copied from one of two silent loci, *mat2* or *mat3* (reviewed in [1]). The system allows investigating multiple facets of recombination, including effects of chromatin structure on recombination and mechanisms of donor choice: how is a particular DNA template selected for recombination when several are available in a cell?

**Figure 1:**
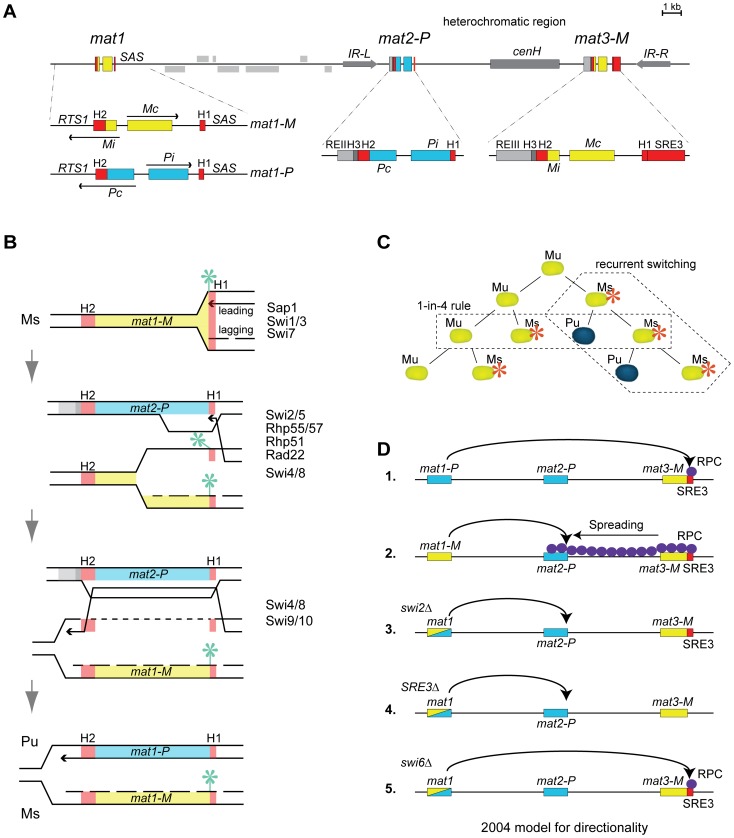
Mating-type region and mating-type switching in *S. pombe*. (A) Schematic representation of the mating-type region showing the expressed, switchable, *mat1* locus and the silent *mat2-P* and *mat3-M* donor loci. Elements are described in the text. (B) Model for the replication-coupled gene conversions of *mat1* responsible for mating-type switching. (C) Pedigree of switching. (D) 2004 model for the directionality of switching ([41]). The model proposes that SRE3 attracts the Swi2/Swi5 recombination complex to the mating-type region. Swi2/Swi5 remains localized near *mat3-M* in P cells, facilitating the use of *mat3-M* as a donor, and spreads over the entire mating-type region in M cells, facilitating the use of *mat2-P*. Use of *mat2-P* is favored over *mat3-M* when Swi2/Swi5 is present at both *mat2-P* and *mat3-M* (as in wild-type M cells) or in the absence of a functional directionality mechanism (as in *SRE3Δ* or *swi2Δ* cells).

The *mat1*, *mat2* and *mat3* loci are linked in the mating-type region (Figure 1). *mat1* determines the mating type of the cell by expressing two divergent regulatory genes, Pi and Pc in P cells (*mat1-P* allele), Mi and Mc in M cells (*mat1-M* allele; [2]). Silent information for the P and M mating types is stored at respectively *mat2* ∼17 kb centromere-distal to *mat1*, and *mat3* ∼29 kb centromere-distal to *mat1* [3]–[5]. The mating-type specific information at *mat1*, *mat2* and *mat3* is flanked by short homology boxes, the centromere-distal H1 box and the centromere-proximal H2 box [2]. Other elements are specific for *mat2* and *mat3* [2],[6],[7] (Figure 1). *mat2* and *mat3* are furthermore embedded in a ∼20 kb heterochromatic domain that spans the *mat2*-*mat3* interval and extends on both sides to inverted repeat boundaries [8],[9]. This domain has been studied extensively. It provides one of the best characterized model systems for how heterochromatic regions can be established and maintained. In this domain, histones are hypoacetylated, histone H3 is methylated at lysine 9 (H3K9me) in an RNA interference-dependent manner, and chromodomain proteins of the HP1 family are associated with the modified histones [8],[10]–[15]. The HP1-like chromodomain protein Swi6 interacts with numerous protein complexes believed to modulate heterochromatin formation, gene silencing and recombination, in ways that remain to a large extent undefined in particular regarding roles in recombination [14],[16]–[19].

Interconversions of the *mat1* locus between *mat1-P* and *mat1-M* lead to mating-type switching (reviewed in [1]). The conversions are coupled to DNA replication which reaches *mat1* from a centromere-distal origin [20],[21]. Switching is initiated by the introduction of a strand-specific imprint in the lagging strand, resulting from the incorporation of two ribonucleotides or a nick between the *mat1* H1 homology box and the mating-type specific information [20],–[28]. In the following rounds of DNA replication, the imprint is placed again on the chromatid made by lagging-strand synthesis, generating a lineage of imprinted, switchable cells [24],[29]. While lagging-strand synthesis propagates the imprinted *mat1* locus in this lineage, leading-strand synthesis produces switched progeny (Figure 1B). At each division, leading-strand synthesis proceeds through the *mat1* H1 homology box and stops at the imprint creating a single-ended double-strand break (DSB) or other recombinogenic molecule with a free 3′end [25],[30]. The free 3′end invades the H1 box of one of the silent loci which is then used instead of *mat1* as template for leading-strand synthesis [29],[31]. This heals the break. Resolution of the recombination intermediate occurs within the H2 homology box with the help of the Swi4/8 and Swi9/10 gene products, producing a switched *mat1* locus [5],[32]–[36]. The newly-switched *mat1* locus does not carry an imprint hence it does not switch at the following S phase, however the chromatid made by lagging-strand synthesis acquires an imprint and starts a new lineage of switchable cells.

A choice of information is made in all switchable cells such that either *mat2-P* or *mat3-M* is used as donor to replicate and convert *mat1*. At this step, *mat2-P* and *mat3-M* are not picked at random. Switchable *mat1-M* cells preferentially use *mat2-P* whereas switchable *mat1-P* cells use *mat3-M* [23],[37],[38]. Coupled with the mechanism of switching outlined above, these directed choices produce a reproducible pattern of mating-type switching where (1) one out of four grand-daughters of a newly-switched cell has a high probability of having a switched mating-type (80–90%; one-in-four rule) and (2) once a cell becomes switchable the probability of recurrent switches in its lineage is very high (80–90%; recurrent-switching rule; Figure 1C).

Previous studies have revealed the importance of donor location and chromatin structure in donor choice [39]–[42]. Strains in which the *mat2* and *mat3* contents were swapped from *mat2-P mat3-M* (*h^90^* configuration) to *mat2-M mat3-P* (*h^09^* configuration) switch inefficiently to the opposite mating-type [39]. Mutations in the chromodomain protein Swi6, the H3K9 methyltransferase Clr4, or the Clr4-complex subunits Clr7 and Clr8 have opposite effects on switching in the *h^09^* and *h^90^* mating-type regions indicating chromatin structure favors use of *mat2* in M cells and use of *mat3* in P cells [39],[42]. The phenotypes of these mutants suggest that unproductive homologous switching occurs in *h^09^* cells where *mat1-M* is converted by *mat2-M* and *mat1-P* by *mat3-P* instead of the productive heterologous switching occurring in *h^90^* cells.

The strand exchange taking place at H1 is likely to be a determining step in donor choice. The Swi2 and Swi5 proteins are believed to facilitate this step together with Rad22, the fission yeast RAD52 homolog [43] and Rhp51, the *S. pombe* RecA homolog [44],[45]. The imprint, detected as a chromosomal fragile site, is formed at *mat1* in *swi2* and *swi5* mutants but a subsequent step in the conversion process fails [5]. Consistent with this step being strand-invasion at the donor loci Swi2 interacts physically with both Swi5 and Rhp51 [17] and Swi5 facilitates Rhp51-mediated strand exchange *in vitro* [46]–[49]. Combined with the observation that Swi2 interacts with Swi6 [17], the properties of Swi2 and Swi5 place these factors close to the point where donor selection takes place.

A model for the directionality of mating-type switching combining effects of chromatin structure and targeted recruitment of recombination proteins was proposed in [41] (Figure 1; referred to as 2004 model below). In this model, the search for a donor starts at *mat2*. If the Swi2/Swi5 recombination-promoting complex (RPC) is encountered at *mat2*, *mat2* is used to convert *mat1*. If RPC is not at *mat2*, the search proceeds to *mat3* and *mat3*, which is constitutively associated with RPC, is used to convert *mat1*. The constitutive association of *mat3* and RPC observed in both P and M cells is proposed to occur through a DNA element named SRE [41] that we will call SRE3 below to reflect its proximity to *mat3*. RPC is localized at SRE3 in P cells – ensuring that *mat3* is used in P cells - but spreads from SRE3 all the way to *mat2* in M cells – ensuring that *mat2* is used in M cells. Spreading of RPC from SRE3 to *mat2* requires Swi6. A recent addition to the model proposes that the spreading is driven by a greater abundance of the Swi2 and Swi5 proteins in M cells resulting from the positive regulation of the *swi2* and *swi5* genes by the M-specific transcription factor Mc [50]. Alternatively, Mc might stimulate the production of a shorter form of Swi2 expressed in P cells through alternative promoter usage [51].

The directionality model summarized above [41] provides a framework for investigations of mating-type switching, although several critical steps in it have no documented mechanism. One unexplained feature is that the search for a donor should start at *mat2*. This step is important because it accounts for *mat2* being used in M cells when Swi2/Swi5 is present at both *mat2-P* and *mat3-M*. The model proposes that a higher-order chromatin structure helps choosing *mat2* by placing it near *mat1* but how this occurs remains unknown. Another aspect of the model that has not been documented experimentally is the physical interaction between SRE3 and Swi2. This is also a crucial element because the model is centered on SRE3 being the sole entry point for Swi2 in the mating-type region, accounting for the observation that Swi2 was not detected in the mating-type region of *SRE3Δ* strains by ChIP [41]. Here, we report further investigations on the directionality of mating-type switching bearing on these and other points. Our results challenge the notions that SRE3 is the sole entry point for Swi2, that Swi2 reaches *mat2* by spreading from SRE3, and that the search for a donor starts at *mat2*. Instead, our results show that directionality requires two recombination enhancers, SRE2 and SRE3, whose ability to stimulate recombination in a cell-type specific manner is not tied to a specific location in the mating-type region. We present evidence that directionality results from competitions between SRE2 and SRE3, governed by cell-type specific chromatin structures.

## Results

### SRE3-independent effects of Swi2 and Swi5 on the directionality of mating-type switching indicate the presence of a second recombination enhancer

The SRE3 element was described as the entry point at which the Swi2/Swi5 complex associates with the mating-type region [41]. Following this initial association, proposed to take place in both cell types, Swi2 could remain at SRE3 in P cells or spread to *mat2* in M cells. Support for this mechanism is provided by ChIP experiments that failed to detect Swi2 anywhere in the mating-type region in strains lacking SRE3 [41]. We examined the model further through a simple genetic test. If the directed association of Swi2 with the mating-type region is abolished in *SRE3Δ* cells the mating-type bias in *SRE3Δ* cells should not be altered by deletion of *swi2*.

**Figure 2:**
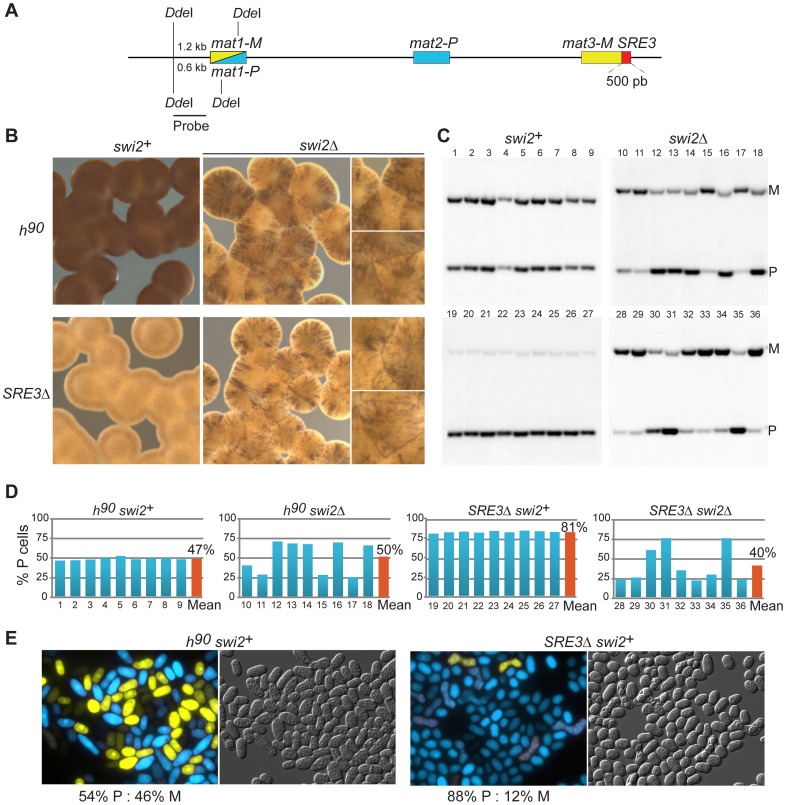
SRE3-independent effects of Swi2 on donor choice. (A) Representation of the mating-type region showing the probe (*Dde*I-*Nsi*I fragment) and restriction sites used for the Southern blots in (C). (B) Iodine staining of sporulated colonies with indicated genotypes. Dark staining reflects efficient switching. Light staining indicates a switching defect that can be either asymmetric towards a preferred mating-type (no staining at colony junctions) or inefficient in both directions (staining at colony junctions). (C) Southern blots of genomic DNA digested with *Dde*I and hybridized to the *Dde*I-*Nsi*I probe shown in (A), for nine independent cultures of *h^90^ swi2* ^+^: 968 (1–9); *h^90^ swi2Δ*: TP133 (10–18); *SRE3Δ swi2* ^+^: TP75 (19–27); *SRE3Δ swi2Δ*: TP157 (28–36). (D) Quantification of *mat1* content estimated from the Southern blots shown in (C). P/(P+M) ratios are plotted as % P cells. (E) Independent measurement of cell type ratios by fluorescence microscopy using a dual reporter system, YFP under control of the M-specific *mfm3* promoter and CFP under control of the P-specific *map2* promoter. M cells are yellow and P cells cyan. *h^90^ swi2* ^+^: TP220; *SRE3Δ swi2* ^+^: TP270. Cell counts from fluorescence microscopy are presented in Table S3. Effects of Swi5 on donor choice are presented in Figure S1. All Southern blot quantifications are summarized in Figure S2.

In *S. pombe*, the efficiency of mating-type switching can be estimated by staining sporulated colonies with iodine vapors. Efficiently-switching strains produce colonies that are stained darkly by iodine vapors because they contain many spores while poorly-switching strains produce lightly-stained colonies [52]. The predominant mating-type in cell populations can be further determined by quantifying the content of *mat1* by Southern blot or PCR. In addition, we developed here a reporter system in which M cell express YFP and P cells express CFP allowing typing individual cells with a fluorescence microscope (Figure 1D). Sporulated *SRE3Δ* colonies were stained lightly by iodine and colonies did not stain at their junctions indicating preferential use of one donor (Figure 2B). Southern blotting, competitive PCR, and fluorescent typing all showed that *SRE3Δ* cells contain predominantly the *mat1-P* allele (P∶M = 82∶18 by Southern blot; P∶M = 88∶12 by cell count; Figure 2; competitive PCR not shown). The *SRE3Δ* strain used for these analyses was made in our laboratory [7] hence these results confirm the observations in [41] with an independent strain and support the conclusion of these authors that *mat2-P* is the preferred donor in *SRE3Δ* cells.

We assessed the effect of deleting *swi2* in both wild-type *h^90^* cells and *SRE3Δ* cells (Figure 2). Iodine staining indicated that deletion of *swi2* severely affected switching efficiency in both backgrounds: *h^90^ swi2Δ* and *SRE3Δ swi2Δ* cells formed streaky colonies staining at their junctions showing that cells are predominantly of the M mating-type in some colonies and predominantly of the P mating type in other colonies (Figure 2B). We measured P∶M ratios in nine independent cultures of respectively *h^90^ swi2^+^*; *h^90^ swi2Δ*; *SRE3Δ swi2^+^*; and *SRE3Δ swi2Δ* cells by Southern blot (Figure 2C). While all *h^90^ swi2^+^* cultures had balanced P∶M ratios and all *SRE3Δ swi2^+^* cultures were predominantly of the P mating-type, large fluctuations in P∶M ratios were observed in *h^90^ swi2Δ* and *SRE3Δ swi2Δ* cultures. The strong phenotypic variability observed in *h^90^ swi2Δ* cultures disagrees with the report [41] that *h^90^ swi2Δ* cells contain predominantly *mat1-P* and that the switching pattern of *h^90^ swi2Δ* cells is indistinguishable from switching in *h^90^ SRE3Δ* cells. Further, the clear phenotypic differences we observed between *SRE3Δ swi2^+^* (P≫M in all colonies; 81% P cells averaged over 9 cultures) and *SRE3Δ swi2Δ* strains (variegated phenotype; 40% P cells averaged over 9 cultures) is not predicted in [41]. Similarly, we observed culture-to-culture variations with, if any, a bias towards M cells in *h^90^ swi5Δ* cultures (72% M cells averaged over 9 cultures; Figure S1) in contrast to [50] who found that *h^90^ swi5Δ* cells are predominantly P. As for the deletion of *swi2^+^*, deletion of *swi5^+^* abrogated the preferential use of *mat2-P* in *SRE3Δ* cells (Figure S1). We conclude that the RPC is necessary for the efficient and preferential choice of *mat2-P* in *SRE3Δ* cells. Since this represents a situation where RPC cannot reach *mat2-P* by spreading from SRE3, this result does not support the spreading model and suggests instead that other DNA element(s) or factors attract Swi2 and Swi5 independently of SRE3.

### Chromosomal deletions adjacent to *mat2-P* impair switching, defining the SRE2 recombination enhancer

**Figure 3:**
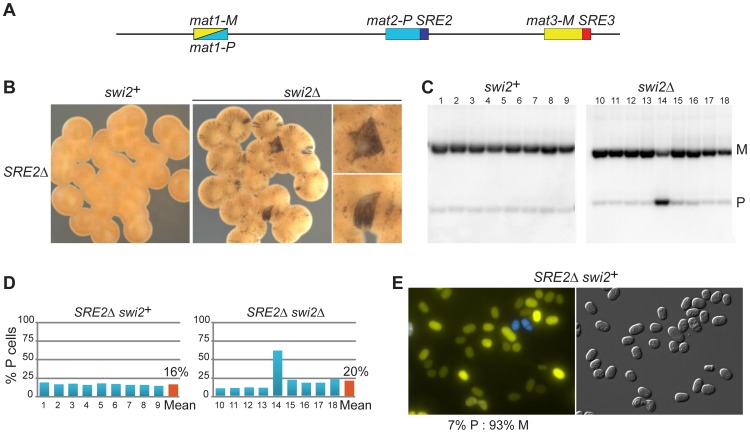
Deletion of SRE2 reduces use of *mat2-P*. (A) Location of SRE2 and SRE3 elements. (B–E) as for Figure 2, with mutant strains lacking SRE2. The strains in (B–D) were: *SRE2Δ swi2* ^+^: TP8; *SRE2Δ swi2Δ*: TP156; the strain in (E) was TP268.

While systematically introducing deletions in the mating-type region we found that a set of nested deletions on the centromere-distal side of *mat2-P* affected switching, defining a ∼500 bp element adjacent to the H1 box, SRE2. Deletion of SRE2 caused a pronounced switching defect (Figure 3). Sporulated *SRE2Δ* colonies were stained very lightly by iodine vapors and they did not stain at their junctions; a Southern blot testing *mat1* content in nine independent cultures indicated a large predominance of M cells in all cultures; and the existence of a strong bias towards M cells was also supported by fluorescence microscopy (Figure 3). Identical phenotypes were obtained when *SRE2Δ* colonies were seeded from P or M spores confirming efficient asymmetric switching favoring *mat3-M* (data not shown). The location of SRE2 relative to *mat2* is similar to the location of SRE3 relative to *mat3* but no extensive sequence similarities were noted between SRE2 and SRE3. Both elements are A-T rich (75% for SRE2 and 72% for SRE3 over 492 bp). The authors of a recent study [51] noticed like us that a deletion adjacent to *mat2-P* prevented efficient use of *mat2-P* however the study did not characterize the element further. Several observations reported below argue against deletion of SRE2 simply preventing use of *mat2* as a donor. They suggest instead that SRE2 regulates donor choice.

As for the strains examined above, deleting *swi2^+^* affected switching in *SRE2Δ* cells. Two types of sporulated colonies were observed following iodine staining, light colonies with a few dark streaks containing mostly M cells, and more rare darker colonies containing a greater proportion of P cells (Figure 3; 80% M cell averaged over nine colonies). Deletion of *swi5* produced a similar phenotype (77% M cell averaged over nine colonies; Figure S1; Southern blot quantifications are summarized in Figure S2.). These phenotypes are consistent with *mat3-M* remaining a preferred donor in *SRE2Δ* cells even when RPC is not present in the cells. This again fails to support the 2004 model, where *mat2-P* is the preferred donor when RPC is not present due to higher-order chromatin structure. Alternatively, SRE2 might be responsible for the higher-order structure postulated by the model.

We investigated the association of Swi2 with parts of the mating-type region by ChIP (Figure S3). In unswitchable *mat1-M* cells, where *mat2-P* is perhaps poised for switching, Swi2 was detected at *mat2-P* and at SRE2 as previously reported [41]. In our experiments, Swi2 was also detected at these locations in *SRE3Δ* cells consistent with an SRE3-independent mode of recruitment to the mating-type region. This recruitment appeared facilitated by SRE2 since the association of Swi2 with *mat2* was reduced in *SRE2Δ* cells (primer pairs 44, 46 and ‘SRE2Δ’ in M cells, Figure S3).

### SRE2 and SRE3 direct donor choice independently of their location

**Figure 4:**
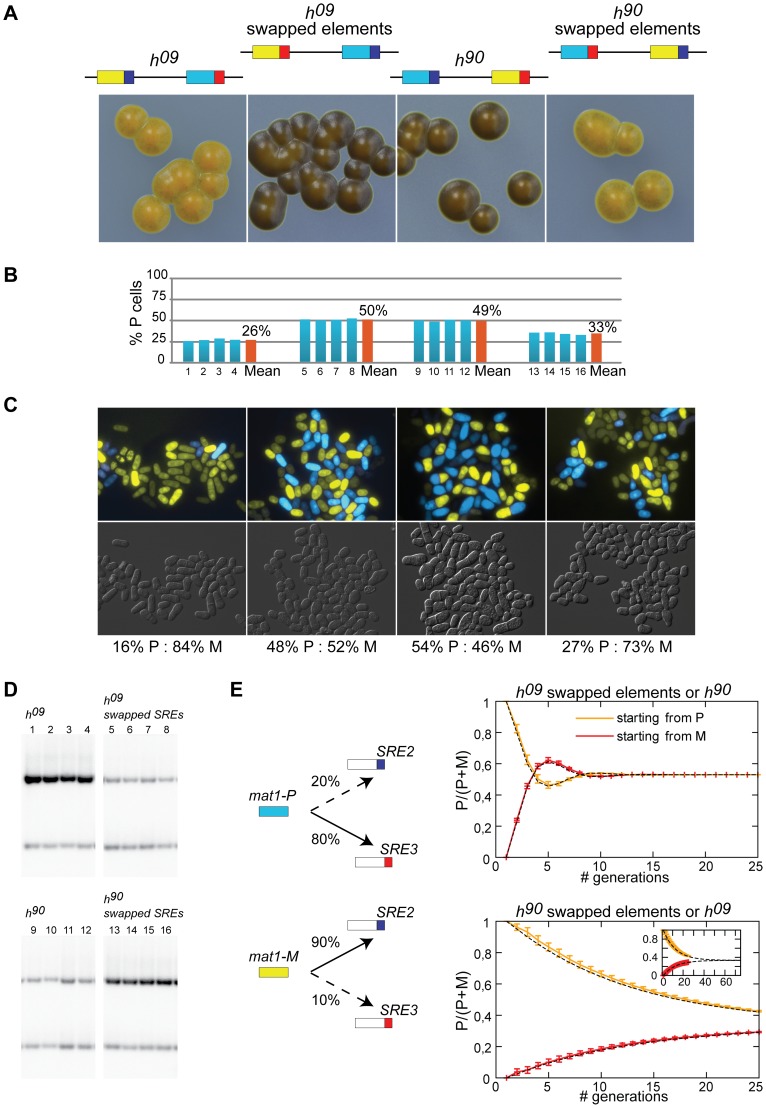
SRE2 and SRE3 mediate directionality ectopically. (A) Schematic representation of *mat2*-*mat3* region and iodine staining of strains with transposed cassette contents or SRE elements. The M mating-type information is in yellow; the P information in light blue; SRE2 in dark blue; SRE3 in red. Light iodine staining indicates poor switching in strains where cassette contents and recombination enhancers are uncoupled. Swapping the recombination enhancers in *h^09^* cells restores switching efficiency to wild-type level. (B) Quantification of *mat1* content as in Figure 2 for four independent cultures of each strain. *h^09^*: PG19 (1–4); *h^09^* with swapped elements: TP38 (5–8); *h^90^*: 968 (9–12); *h^90^* with swapped elements: TP39 (13–16). (C) Cell-type quantification by fluorescence microscopy as in Figure 2E. The strains were, from left to right: TP262, TP263, TP220, TP265. (D) Blots used for the quantifications in (B). (E) Computer simulations of P/(P+M) ratios in growing cultures started from P or M cells, when SRE2 and SRE3 are chosen at the rates indicated on the left. A small difference in the rates at which SRE2 and SRE3 are ‘wrongly’ used (dotted lines; 20% and 10% respectively) leads to unbalanced mating-type ratios and might contribute to the biases observed in *h^09^* cells and *h^90^* cells with swapped elements.

A deletion reducing the use of a donor cassette is not on its own evidence that the deletion removed a directionality element. We explored the possibility that SRE2 and SRE3 are genuine directionality elements by engineering *h^09^* cells. The donor loci are *mat2-M mat3-P* in the *h^09^* mating-type region [39]. The cassette contents are precisely exchanged between the H2 and H1 homology boxes placing *mat2-M* near SRE2 and *mat3-P* near SRE3. This arrangement results in inefficient switching to the opposite mating-type ([39]; Figure 4). The *h^09^* strain provides a useful tool to study directionality since it allows designing experiments in which the tested outcome is improved switching rather than loss of switching. We tested whether swapping SRE2 and SRE3 in *h^09^* cells improved heterologous switching.

Figure 4 shows that *h^09^* cells with swapped elements switched mating-type very efficiently and produced populations with equal proportions of P and M cells. Their sporulated colonies were undistinguishable from *h^90^* colonies. Their *mat1* content examined by Southern blot was evenly balanced and fluorescence microscopy confirmed equal proportions of P and M cells in colonies (Figure 4). Conversely *h^90^* cells with swapped SRE elements switched mating-type poorly, produced mainly *mat1-M* cells as *h^09^* cells with unswapped elements do, and formed colonies very similar to *h^09^* colonies (Figure 4). Together these experiments show that the *PSRE2 MSRE3* combination (whether *mat2-PSRE2 mat3-MSRE3* in wild-type *h^90^* cells with native elements or *mat2-MSRE3 mat3-PSRE2* in *h^09^* cells with swapped elements) leads to balanced use of the two cassettes while the *PSRE3 MSRE2* combination (whether *mat2-MSRE2 mat3-PSRE3* in *h^09^* cells with native elements or *mat2-PSRE3 mat3-MSRE2* in *h^90^* cells with swapped elements) leads to inefficient heterologous switching. We conclude from these observations that SRE2 and SRE3 behave as directionality elements responsible for the balanced heterologous switching observed in *h^90^* cells. P cells select the cassette adjacent to SRE3 while M cells select the cassette adjacent to SRE2 and SRE2 and SRE3 can both be recognized ectopically when their location relative to *mat1* has been swapped.

### Asymmetries

Should SRE2 and SRE3 be the sole determinants of directionality and should their action be fully symmetrical, *h^09^* cells with native SRE elements and *h^90^* cells with swapped SRE elements would engage in futile cycles where *mat1-P* selects *mat3-PSRE3* (in *h^09^*) or *mat2-PSRE3* (in *h^90^* cells with swapped elements) and *mat1-M* selects *mat2-MSRE2* (in *h^09^*) or *mat3-MSRE2* (in *h^90^* cells with swapped elements; Figure 4). Two types of colonies would be formed, one type containing predominantly P cells, the other predominantly M cells. This is not what is observed. Both *h^09^* cells with native elements and *h^90^* cells with swapped elements form populations where M cells predominate (Figure 4) indicating preferential choice of the cassette adjacent to SRE2. The fact that the bias is towards the *MSRE2* cassette in both cases even though the *MSRE2* cassette occupies different locations in the two strains shows that a preponderant cause for the bias is location independent.

The mechanisms responsible for directionality are likely to fail occasionally, allowing the ‘wrong’ donor to be selected. We reasoned that a small error rate would not have a strong impact on the overall composition of *h^90^* cell populations, but the same error rate could have more profound consequences in *h^09^* populations because the mistakes would lead to changes in mating-type that would subsequently be stably propagated through homologous switching. We modeled a situation where P cells use predominantly SRE3 to select a donor for switching, while M cells select predominantly SRE2 (Figure 4). We allowed a low occurrence of mistakes in both cell types, where P cells occasionally use SRE2 (20% of attempted switches) while M cells use SRE3 more rarely (10% of attempted switches). As expected such a bias leads to an accumulation of M cells in both *h^09^* cells with native elements and *h^90^* cells with swapped elements supporting the hypothesis that aberrant choices contribute to the preponderance of M cells in these strains. We note that in addition to SRE2 being more promiscuous than SRE3, the cassette content in the *MSRE2* combination might facilitate use of *MSRE2* over *PSRE3* in P cells.

### SRE2 and SRE3 enhance recombination in both cell types

**Figure 5:**
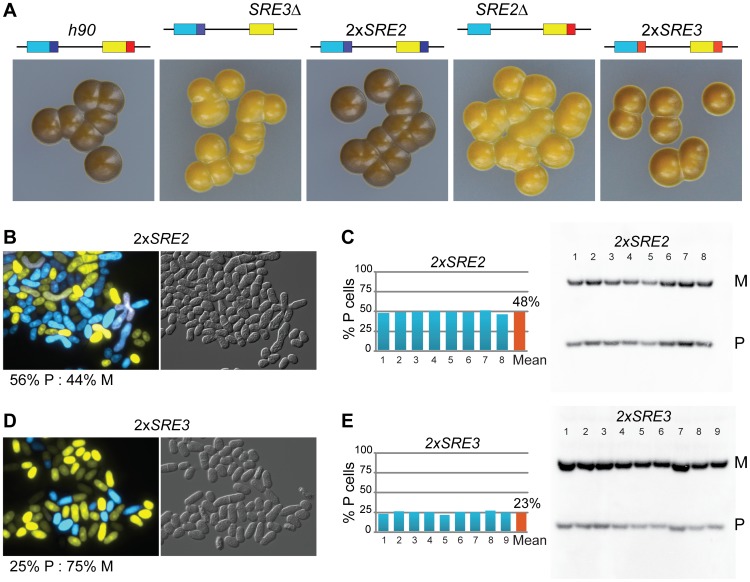
SRE2 and SRE3 can stimulate recombination in both cell types. (A) Iodine staining of strains with the indicated mating-type regions (M: yellow; P: light blue; SRE2: dark blue; SRE3: red) shows that SRE2 stimulates recombination at *mat3* when it is substituted for SRE3 (2×SRE2 strain) and that SRE3 stimulates recombination at *mat2* when it is substituted for SRE2 (2×SRE3 strain), albeit less efficiently. The strains were, from left to right: SP837, TP75, TP126, TP8, TP303. (B) Fluorescence microscopy and (C) quantification of *mat1* content by Southern blot confirm balanced donor use in 2×SRE2 cells showing that P cells use SRE2 (TP273 and TP126 strains). (D–E) Same analyses as (B–C) for 2×SRE3 (TP313 and TP303 strains) indicate that M cells are not fully proficient in the use of SRE3 and accumulate in 2×SRE3 populations.

As a way of testing the extent to which P cells can use SRE2 we replaced SRE3 with SRE2 (*mat2-PSRE2 mat3-MSRE2* strain referred to as 2×SRE2). The 2×SRE2 strain switched mating-type efficiently as judged from its dark iodine staining and balanced ratio of P and M cells (Figure 5). 2×SRE2 populations contained 48% P cells according to Southern blot, 56% P cells according to microscopy. The phenotype of the 2×SRE2 strain shows that P cells are proficient in the use of the SRE2 element in *mat3-MSRE2* otherwise P cells would accumulate in the population of 2×SRE2 cells. To illustrate this further *SRE3Δ* colonies are shown near the 2×SRE2 strain for comparison in Figure 5. SRE2 at *mat3-M* considerably improves heterologous switching showing that P cells use SRE2. Even though 2×SRE2 cells switch mating-type efficiently, mating-type selectivity in 2×SRE2 is not as in wild-type leading us to propose that donor choice is randomized in 2×SRE2 rather than directional. A differential behavior of 2×SRE2 and wild-type mating-type region is shown for example in the next section where *h^90^ swi6Δ* and *2×SRE2 swi6Δ* strains clearly differ from each other. Similarly we replaced SRE2 with SRE3 (*mat2-PSRE3 mat3-MSRE3* strain referred to as 2×SRE3). The 2×SRE3 strain produced a mixture of P and M cells which shows that M cells can use SRE3, however with a bias towards M cells (Figure 5). 2×SRE3 populations contained 23% P cells as estimated from Southern blot, 25% estimated from microscopy. Together with the results presented above for the 2×SRE2 strain these ratios indicate that M cells are not as proficient at using *mat2-PSRE3* as P cells are at using *mat3-MSRE2*. In summary SRE2 can stimulate recombination of donor loci with *mat1* efficiently not only in M cells but also in P cells whereas SRE3 is more active in P cells than in M cells. The ability of each element to function in both cell types shows that these elements are not strictly dependent on cell-type-specific factors to stimulate recombination.

### Effects of chromatin — mechanisms of recombination enhancement by SRE2 and SRE3

A remarkable aspect of mating-type switching is that the donor loci are in heterochromatin. We asked whether and how the ability of the SRE elements to stimulate recombination was affected by heterochromatin through epistasis analyses using cells lacking the chromodomain protein Swi6.

**Figure 6:**
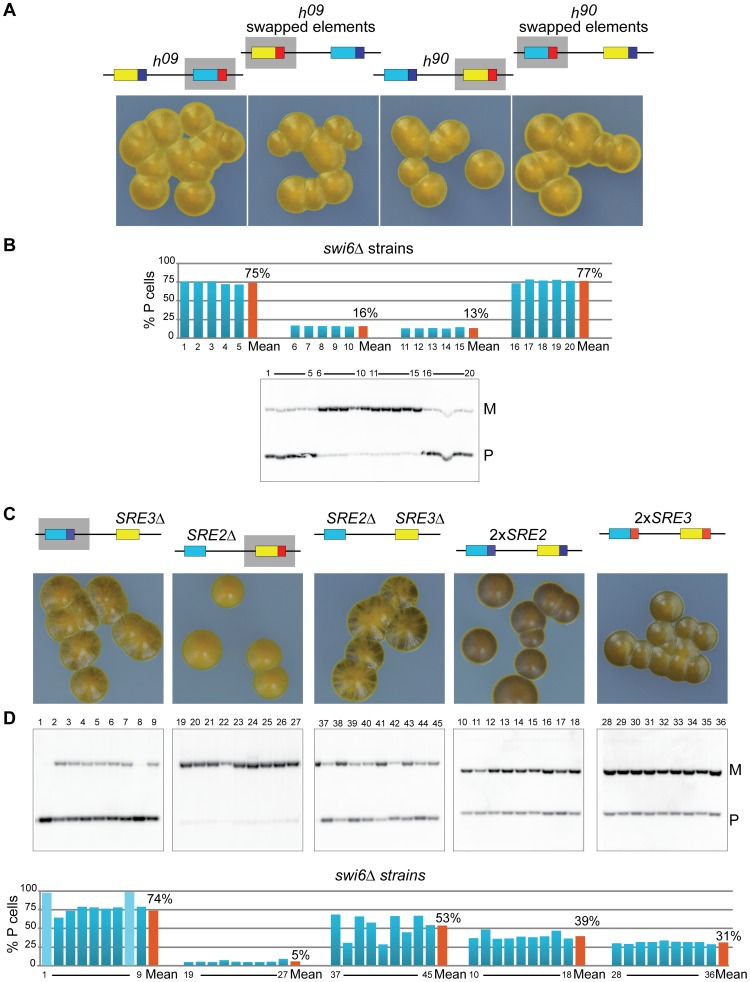
Chromatin structure regulates the relative efficiency of the SRE2 and SRE3 recombination enhancers. (A–B) Same analysis as in Figure 4A–B, for *swi6Δ* strains. Grey boxes indicate preferred donors according to Southern blot quantification. SRE3 is preferred over SRE2 independently of cassette content and location in the four *swi6Δ* strains examined. A comparison with the *swi6* ^+^ strains displayed in Figure 4A–B shows the overriding effect of Swi6 on enhancer choice. (C–D) Same analysis as in Figure 5, for *swi6Δ* strains. SRE2 and SRE3 can each promote recombination in *swi6Δ* strains. When only one enhancer is present, or when two enhancers of the same kind are present, Swi6 has little effect on donor choice. Isolate 1 and 8 for the *SRE3Δ swi6Δ* strain had rearranged mating-type regions and were not taken into account for the analysis.

Deletion of *swi6^+^* in *h^09^* or *h^90^* cells with native or swapped elements radically altered donor choice (compare Figure 4 and 6). Populations of *h^90^* cells or *h^09^* cells with swapped elements went from balanced P∶M ratios (49% and 50% P resp.) to containing predominantly M cells (87% and 84% resp.). Conversely the M bias in populations of *h^09^* cells or *h^90^* cells with swapped elements was abrogated by *swi6Δ*. In all cases, the changes reflected that use of the cassette adjacent to SRE2 was greatly decreased in favor of the cassette adjacent to SRE3 following deletion of *swi6^+^*, as indicated in Figure 6. These phenotypes show that Swi6 biases donor choice towards the cassette controlled by SRE2, or away from the cassette controlled by SRE3, whether the cassette contains the P or M information, and whether it is located at *mat2* or *mat3*.

Reduced selection of SRE2 in *h^90^ swi6Δ* cells depended on the presence of SRE3 in the same cells. No change in preferred donor was observed in *h^90^ SRE3Δ* cells following deletion of *swi6^+^*, SRE2 kept being used (compare *SRE3Δ* in Figure 2 to *SRE3Δ swi6Δ* in Figure 6; *mat1-P* predominates in both). This indicated that SRE2 could stimulate recombination at *mat2-PSRE2* in the absence of Swi6 when SRE3 was not present. Very inefficient switching in *SRE2Δ SRE3Δ swi6Δ* cells confirmed that the selection of *mat2-PSRE2* in *SRE3Δ swi6Δ* cells depended on SRE2 (Figure 6; inefficient switching in the *SRE2Δ SRE3Δ swi6Δ* strain produces colonies staining at their junctions and large fluctuations in P/M ratios). Similarly, use of *mat3-MSRE3* in *SRE2Δ swi6Δ* cells required SRE3 (compare *SRE2Δ swi6Δ* with *SRE2Δ SRE3Δ swi6Δ* in Figure 6). In summary these phenotypes show that both SRE2 and SRE3 can stimulate recombination in the absence of Swi6. Competitions between the two enhancers drive donor selection both in the absence and presence of Swi6. In the absence of Swi6 SRE3 is preferred over SRE2. When present, Swi6 biases donor selection towards SRE2.

## Discussion

Some forms of recombination occur with an extraordinary efficiency in heterochromatin as illustrated by fission yeast mating-type switching. In mating-type switching, a euchromatic locus, *mat1*, undergoes productive recombinogenic interactions with a heterochromatic partner in every other dividing cell. Not only are these recombination events frequent, they are also exquisitely fine-tuned such that a specific donor is selected for each conversion of *mat1*. Our work identifies small, portable, DNA elements responsible for donor choice and provides new insights into the mechanisms responsible for the directionality of switching. Some of our observations differ from previous reports [41],[50]. We discuss here these discrepancies and use our findings to build a new model for the directionality of mating-type switching.

### The SRE elements direct donor choice ectopically

Cells in which the silent-cassette contents are swapped (*h^09^*) switch mating-type inefficiently, indicating cells fail to choose heterologous donors when the donors are not at their endogenous location [39]. Here, we find that a crucial aspect of donor location is proximity of the donors to their respective recombination enhancers, SRE2 and SRE3. The determining role of SRE2 and SRE3 in donor selection was revealed by the high efficiency of switching in *h^09^* cells when the SRE elements were swapped concomitantly with the contents of *mat2* and *mat3* (Figure 4). Heterologous donors could be found efficiently even when they were not at their endogenous location, provided the coupling with their cognate recombination enhancers was maintained.

The fact that *h^09^* cells with swapped SRE elements switch well has strong implications for the 2004 model. The 2004 model is a two-component model integrating effects of donor positioning relative to *mat1* (in the model the recombinogenic DSB at *mat1* encounters *mat2* before it encounters *mat3*) and presence of RPC (the first RPC-associated donor encountered is used; Figure 1). In *h^09^* cells with swapped elements a search starting at *mat2* would encounter SRE3 first. SRE3 being the proposed nucleation site for RPC, constitutively associated with RPC in both cell types, *mat2-MSRE3* should be selected preferentially in both cell types which is clearly not the case. Our observations show instead that M cells choose SRE2 and P cells choose SRE3 when these elements are present, independently of their location.

One way of reconciling the portability of the SRE elements with the 2004 model is to propose that SRE2 is responsible both for the higher-order chromatin structure that brings *mat2* close to *mat1* in this model and also for directing the spreading of Swi2 away from SRE3 in M cells. While such roles for SRE2 should be envisioned and tested, other observations we made suggest that Swi2 does not reach *mat2* by spreading from SRE3.

### RPC catalyzes asymmetric switching in situations where RPC was not previously detected by ChIP and in the absence of SRE3

ChIP experiments reported in previous publications have detected large, cell-type specific variations in the association of RPC with the mating-type region [41],[50]. RPC was detected over the entire *mat2*-*mat3* interval in M cells but the association was restricted to SRE3 in P cells [41]. In cells lacking SRE3, RPC was not detected at all [41]. While these strikingly differential associations hint to some relevance for directionality, how the associations lead to the selection of a specific donor is not straightforward. RPC associations do not on their own determine which cassette will be used since the association of RPC with SRE3 is cell-type independent. Here, we found that RPC catalyzes switching even in situations where RPC was not previously detected by ChIP [41] and in the absence of SRE3. In our experiments, the pronounced bias towards the P mating-type displayed by *SRE3Δ* cells was abolished in *SRE3Δ swi2Δ* cells and in *SRE3Δ swi5Δ* cells, showing RPC is necessary for the preferential use of *mat2-P* in *SRE3Δ* cells (Figure 2 and Figure S1). Not only is this epistatic relationship not predicted by the 2004 model – the model predicts that the *SRE3Δ swi2Δ* double mutant should switch like *SRE3Δ* – but the 2004 model specifically relies on *swi2Δ* and *SRE3Δ* cells having identical phenotypes, which is also contradicted by our results (Figure 2).

Based on our genetic evidence we suggest that ChIP has failed to detect interactions between Swi2 and the mating-type region that are relevant to directionality. Difficulties in detecting the association of Swi2 with the mating-type region might be due to the fact that Swi2 is not an abundant protein, that relevant interactions occur in a short window of the cell cycle, or to the fact that ChIP experiments have been conducted in switching-defective cells lacking elements at *mat1* that might participate in directionality as indicated in [24]. Unlike [41], we observed that in M cells Swi2 remained associated with *mat2-P* and SRE2 in the absence of SRE3 (Figure S3). A core feature in the 2004 model is that Swi2 spreads from SRE3 to *mat2* in P cells. Spreading of a protein along the chromatin fiber can be difficult to distinguish from other mechanisms that might lead to the same final associations. Binding at multiple sites might give the appearance of spreading from one of the sites. Here, we suggest that Swi2 does not have to spread from SRE3 to facilitate switching at SRE2.

### Effects of chromatin on the ability of SRE2 and SRE3 to stimulate recombination

We observed that both SRE2 and SRE3 can stimulate recombination in the absence of Swi6. While populations of *SRE2Δ swi6Δ* cells were predominantly M and populations of *SRE3Δ swi6Δ* cells were predominantly P these biases were lost in populations of *SRE2Δ SRE3Δ swi6Δ* cells (Figure 6C–D) showing SRE3 stimulates recombination with *mat3-M* in *SRE2Δ swi6Δ* cells and SRE2 stimulates recombination with *mat2-P* in *SRE3Δ swi6Δ* cells. We furthermore observed that competitions between SRE2 and SRE3 take place in *swi6Δ* cells when both elements are present. While *SRE3Δ swi6Δ* populations were predominantly P (Figure 6C–D), reflecting choice of SRE2, *h^90^ swi6Δ* populations were predominantly M (Figure 6A–B), reflecting choice of SRE3, from which we conclude that SRE3 outcompetes SRE2 in *h^90^ swi6Δ* cells. The switching phenotypes of *h^09^ swi6Δ*; *h^09^* with swapped elements *swi6Δ*; and *h^90^* with swapped elements *swi6Δ* cells all show that SRE3 is preferred over SRE2 in *swi6Δ* cells when both elements are present (Figure 6A–B).

Swi6 exerts major effects on mating-type switching through SRE2 and SRE3. Comparing Figure 4 and Figure 6A–B shows that Swi6 tilts the relative efficiency of the two elements, allowing SRE2 to be preferred over SRE3 in M cells. We suggest that this effect is key to directionality. Several lines of evidence have established that heterochromatin differs in the mating-type region of P and M cells making heterochromatin a good candidate for providing cell-type specificity in mating-type switching. Ectopic reporters are more strongly repressed in M cells than in P cells, whether the reporters are near *mat2* or *mat3* [7],[53], (G. Thon unpublished data) and consistently more Swi6 is detected over the entire *mat2*-*mat3* region in M cells than in P cells [8]. These differences between P and M cells are likely to reflect differential associations of various protein complexes over the entire mating-type region, including but not limited to Swi6, Swi2 and Swi5 [14],[16]–[19],[54]. Global changes over the entire region would account for our observation that the effects of Swi6 on SRE2 and SRE3 were independent of donor location (Figure 6). The model for the directionality of switching outlined below proposes that differences in the chromatin structure of P and M cells determine which recombination enhancer is used in each cell type.

### Model for the directionality of mating-type switching

**Figure 7:**
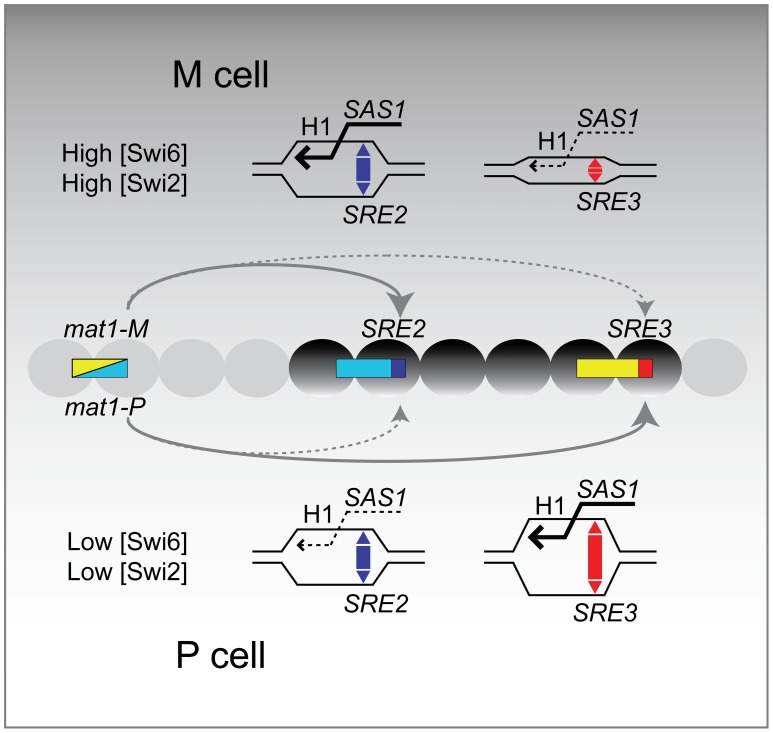
Model for the directionality of mating-type switching. The SRE2 and SRE3 elements promote recombination at their adjacent cassettes. The relative efficiency of the two elements is affected by cell-type specific differences in chromatin structure. SRE3 is more efficient than SRE2 in P cells while SRE2 is more efficient in M cells. Mechanistically, SRE2 and SRE3 might exert these effects by tethering their adjacent cassette to *mat1* in a cell-type specific manner (not depicted here) or they might facilitate D-loop formation at their adjacent H1 box, facilitating strand invasion by the recombinogenic *mat1* DNA strand as discussed in the text. The drawing indicates that SRE3 accessibility is reduced in M cells compared with P cells, but our data do not distinguish between reduced accessibility of SRE3 and increased accessibility of SRE2. ‘High’ and ‘Low’ refer to the local abundance of Swi2 and Swi6 in the mating-type region.

We propose a simple model for the directionality of mating-type switching that takes into account our observations (Figure 7). This model is an alternative to models where the recombination enhancers favor cell-type specific interactions between the donor loci and *mat1* through DNA looping but it is not incompatible with looping models.

In the proposed model SRE2 and SRE3 compete to capture the free DNA end generated at *mat1*. When Swi6 and associated factors are in comparatively low abundance in the mating-type region as is the case in P cells, SRE3 stimulates recombination at its adjacent H1 homology box more efficiently than SRE2. When Swi6 and associated factors are in greater abundance in the mating-type region, as is the case in M cells, SRE2 is more efficient than SRE3.

Several mechanisms can be envisioned for how SRE2 and SRE3 might facilitate strand invasion at their adjacent H1 box in a chromatin-dependent manner. SRE2 and SRE3 might have an intrinsic ability to facilitate D-loop formation as suggested by their low melting temperature (predicted from 72–75% AT content and data not shown). Indeed, evidence has been presented that SRE2 can form a heteroduplex with DNA adjacent to the *mat1* H1 box [33]. Swi6 could modulate the ability of SRE2 and SRE3 to stimulate strand-invasion at H1 through changes in chromatin structure. Swi6 binds to nucleosomes methylated at H3K9 and it oligomerizes. The association of Swi6 with chromatin *per se* might constrain the topology of DNA around H1 and the SRE elements in a way that would alter D-loop induction by the SRE elements and depend on the concentration of Swi6. Other, non mutually-exclusive effects of SRE2 and SRE3 could be through the positioning of nucleosomes. Swi6 might induce the local sliding or displacement of nucleosomes through one of its associated ATP-dependent chromatin remodeling complexes (RSC, Ino80, FACT; [18],[19]) thereby altering the ability of a recombination enhancer to increase H1 accessibility. Finally, direct interactions might take place between the recombination enhancers and recombination factors such as Swi2 as suggested in the case of Swi2 and SRE3 [41]. Directionality would occur if SRE3 had a higher affinity for Swi2 than SRE2 but a lower peak efficiency than SRE2 when stimulating recombination in the context of heterochromatin. At low concentration of Swi2, SRE3 but not SRE2 would be associated with Swi2, promoting invasion of its adjacent cassette. At high concentrations of Swi2, SRE2 would not only be associated with Swi2 but it would use its associated Swi2 more efficiently than SRE3, leading to preferred choice of SRE2 over SRE3. Low association of Swi6 and Swi2 in the mating-type region of P cells would promote invasion of the SRE3-adjacent cassette. High association of Swi6 and Swi2 in the mating-type region of M cells would promote invasion of the SRE2-adjacent cassette.

How pre-existing chromatin structures affect recombination and DSB repair is poorly understood in spite of a great relevance for the maintenance of genome integrity in all eukaryotes. Competitions between donors for gene conversions [55],[56] and regional, cell-type specific, control of recombination [57]–[59] were observed in *S. cerevisiae* similar to what we observed here. Indeed, much of our knowledge on the effects of chromatin on recombination was acquired using *S. cerevisiae* [59]–[61]. Our characterization of the fission yeast SRE elements opens the field for further *in vivo* and *in vitro* studies of recombination regulation in other chromatin contexts.

## Materials and Methods

Standard procedures were used to construct and examine *S. pombe* strains. The details of the strain constructions, Southern blots and microscopy are presented in Text S1 (Extended experimental procedures). Strain genotypes are listed in Table S1 and oligonucleotide sequences in Table S2.

## Supporting Information

Figure S1 Effects of Swi5 on donor choice. The content of *mat1* was estimated by quantifying. Southern blots as in Figure 2, for nine independent cultures of the indicated strains. (A) shows that deletion of *swi5* ^+^ results in culture-to-culture fluctuations, with a general bias towards M cells. The strains were *h^90^ swi5* ^+^: 968 (1–9); *h^90^ swi5Δ*: TP138 (10–18). (B) shows that deletion of *swi5^+^* abrogates the preferential use of *mat2-P* in *SRE3Δ* cells. The strains were *SRE3Δ swi5* ^+^: TP75 (1–9); *SRE3Δ swi5Δ*: TP150 (10–18). (C) shows that deletion of *swi5* ^+^ causes some culture-to-culture variation in *SRE2Δ* cells, with a general bias towards M cells. The strains were *SRE2Δ swi5* ^+^: TP8 (1–9); *SRE2Δ swi5Δ*: TP149 (10–18).
(TIF)

Figure S2 Summary of *mat1* content in wild-type and mutant strains.
(TIF)

Figure S3 Effects of SRE2 and SRE3 on the association of Swi2 with the mating-type region. The association of Swi2 with the mating-type region was assayed using strains with a *13myc* tag at the C-terminus of Swi2. (A) Schematic overview of the silent mating-type region indicating where Swi2 binding was measured. (B) Quantification of ChIP experiments performed with stable P (*mat1-PΔ17::LEU2*; upper panel) or M (*mat1-Msmt-0*; lower panel) strains harboring *SRE2Δ* or *SRE3Δ* as indicated. The primer pairs used were as in [41] except for primers at *SRE2Δ* and *SRE3Δ*, designed to replace respectively primer pair 46 and 69. Enrichments of Swi2 in the regions of interest were calculated relative to *act1*. For these regions, the distribution of Swi2 in wild-type cells was similar to previously published data [41] with a globally more pronounced association of Swi2 in M cells than in P cells. Unlike [41], Swi2 was detected at *mat2* in M cells (primers: 44, 46) in both the presence and absence of SRE3. This indicates that Swi2 can be attracted to the mating-type region independently of SRE3. The association of Swi2 with *mat2* depended on SRE2 (primers: 44, SRE2Δ). (C) Pictures of gels quantified in B. Non-saturated images were used for the quantification. The P strains were: WT: SPA327; *SRE2Δ*: TP366; *SRE3Δ*: TP197. The M strains were: WT: TP186; *SRE2Δ*: TP367; *SRE3Δ*: TP192.
(TIF)

Table S1 Strain table.
(DOC)

Table S2 Oligonucleotide sequences.
(DOCX)

Table S3 Cell counts from fluorescence microscopy.
(DOCX)

Text S1 Extended experimental procedures.
(DOC)
