# Supplementary figures and images for: Two Portable Recombination Enhancers Direct Donor Choice in Fission Yeast Heterochromatin
Source: PLoS Genet. 2013 Oct 24;9(10):e1003762. doi: 10.1371/journal.pgen.1003762 (PMC3812072; doi:10.1371/journal.pgen.1003762)

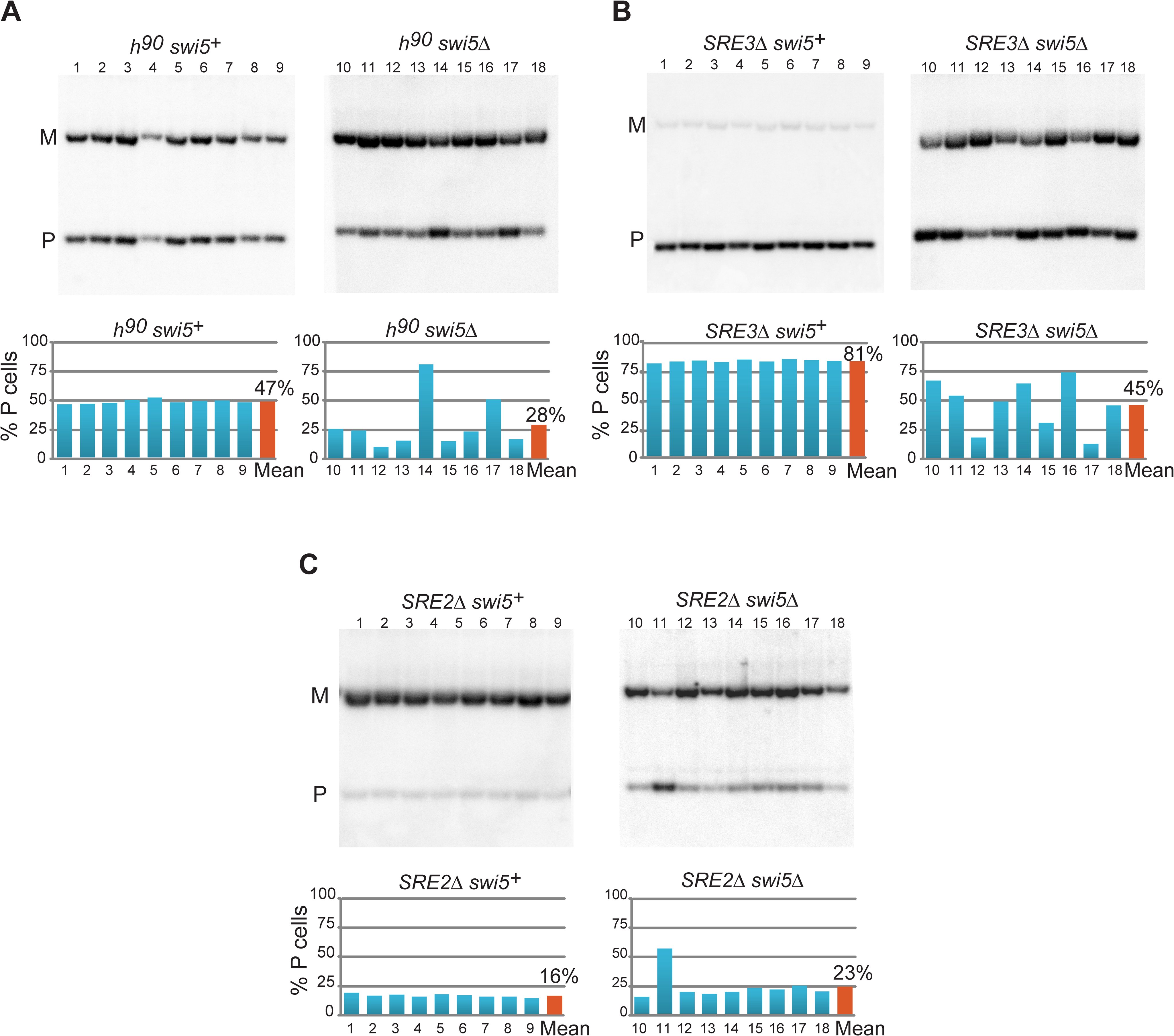

Supplement: Figure S1 — Effects of Swi5 on donor choice. The content of mat1 was estimated by quantifying. Southern blots as in Figure 2, for nine independent cultures of the indicated strains. (A) shows that deletion of swi5 + results in culture-to-culture fluctuations, with a general bias towards M cells. The strains were h90 swi5 +: 968 (1–9); h90 swi5Δ: TP138 (10–18). (B) shows that deletion of swi5+ abrogates the preferential use of mat2-P in SRE3Δ cells. The strains were SRE3Δ swi5 +: TP75 (1–9); SRE3Δ swi5Δ: TP150 (10–18). (C) shows that deletion of swi5 + causes some culture-to-culture variation in SRE2Δ cells, with a general bias towards M cells. The strains were SRE2Δ swi5 +: TP8 (1–9); SRE2Δ swi5Δ: TP149 (10–18). (TIF) [file pgen.1003762.s001.tif]

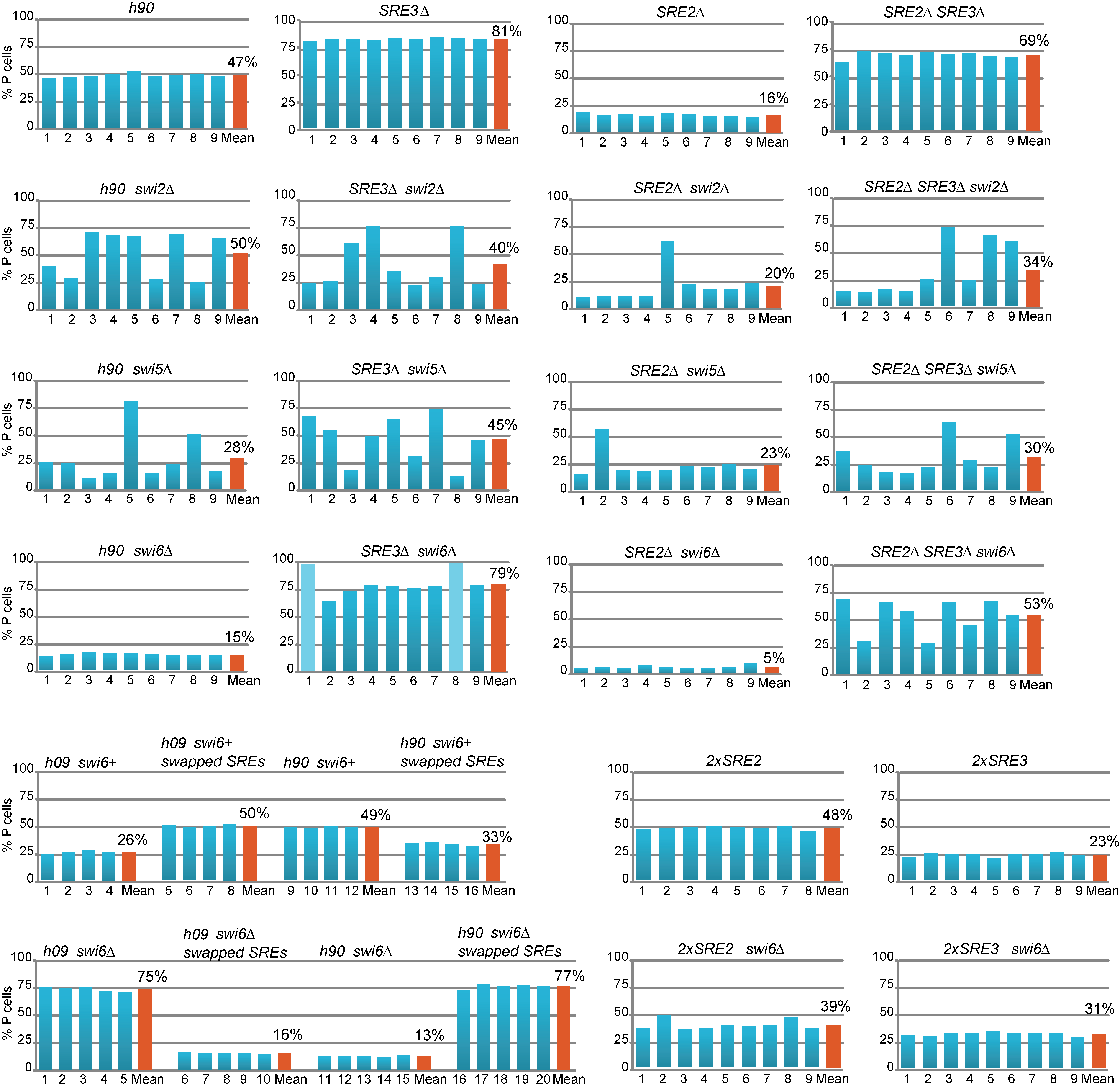

Supplement: Figure S2 — Summary of mat1 content in wild-type and mutant strains. (TIF) [file pgen.1003762.s002.tif]

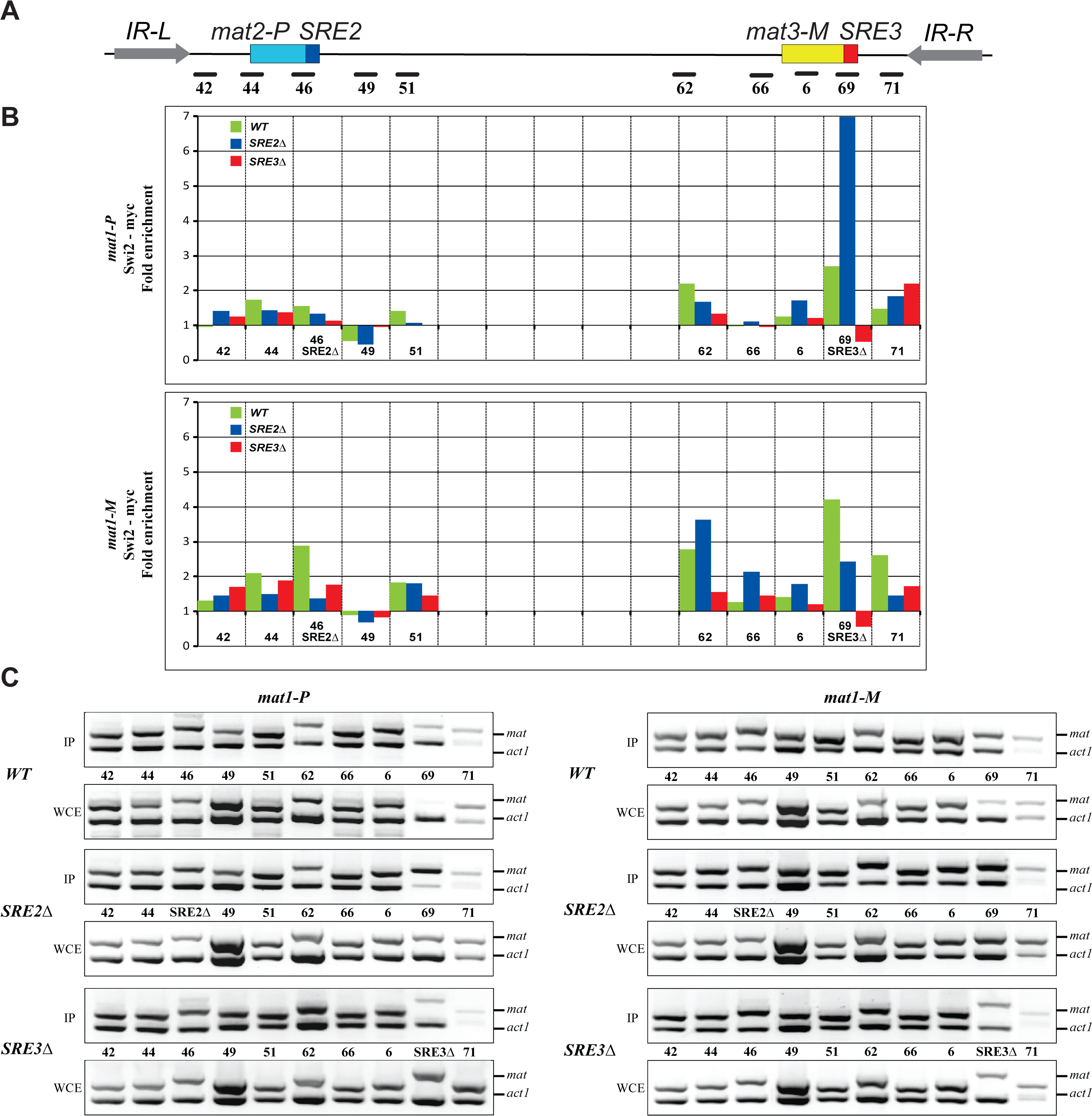

Supplement: Figure S3 — Effects of SRE2 and SRE3 on the association of Swi2 with the mating-type region. The association of Swi2 with the mating-type region was assayed using strains with a 13myc tag at the C-terminus of Swi2. (A) Schematic overview of the silent mating-type region indicating where Swi2 binding was measured. (B) Quantification of ChIP experiments performed with stable P (mat1-PΔ17::LEU2; upper panel) or M (mat1-Msmt-0; lower panel) strains harboring SRE2Δ or SRE3Δ as indicated. The primer pairs used were as in [41] except for primers at SRE2Δ and SRE3Δ, designed to replace respectively primer pair 46 and 69. Enrichments of Swi2 in the regions of interest were calculated relative to act1. For these regions, the distribution of Swi2 in wild-type cells was similar to previously published data [41] with a globally more pronounced association of Swi2 in M cells than in P cells. Unlike [41], Swi2 was detected at mat2 in M cells (primers: 44, 46) in both the presence and absence of SRE3. This indicates that Swi2 can be attracted to the mating-type region independently of SRE3. The association of Swi2 with mat2 depended on SRE2 (primers: 44, SRE2Δ). (C) Pictures of gels quantified in B. Non-saturated images were used for the quantification. The P strains were: WT: SPA327; SRE2Δ: TP366; SRE3Δ: TP197. The M strains were: WT: TP186; SRE2Δ: TP367; SRE3Δ: TP192. (TIF) [file pgen.1003762.s003.tif]
