# Supplementary material for: Two Portable Recombination Enhancers Direct Donor Choice in Fission Yeast Heterochromatin
Source: PLoS Genet. 2013 Oct 24;9(10):e1003762. doi: 10.1371/journal.pgen.1003762 (PMC3812072; doi:10.1371/journal.pgen.1003762)
Supplement: Table S1 — Strain table. (DOC) [file pgen.1003762.s004.doc]

**Table S1. Strain table.**

| **Strain** | **Mating-type region** | **Other** |
| --- | --- | --- |
| **968** | *h^90^* |  |
| **PG9** | *mat3-M(EcoRV)::ura4* | *ura4-D18 leu1-32 ade6-216* |
| **PG19** | *h^09^* | *ura4-D18 leu1-32 ade6-210* |
| **PG3089** | *mat3-M(EcoRV)::ura4* | *clr4::LEU2 ura4-D18 leu1-32 ade6-210* |
| **SpA327** | *mat1-PD17::LEU2* | *swi2::13myc::kanR leu1-32 ade6-210* |
| **SP837** | *h^90^* | *ura4-D18 leu1-32 ade6-216* |
| **TP1** | *L(SacI)::ade6 K(XbaI)::ura4* | *clr3Δ::kanR ura4-DS/E leu1-32 ade6-210* |
| **TP2** | *mat2-PΔSRE2(460bp)* | *clr3Δ::kanR ura4-DS/E leu1-32 ade6-210 arg-* |
| **TP5** | *mat2-PΔSRE2(859bp)* | *clr3Δ::kanR ura4-DS/E leu1-32 ade6-210 arg-* |
| **TP8** | *mat2-PΔSRE2(859bp)* | *ura4-D18 leu1-32 ade6-210* |
| **TP20** | *mat3-M(EcoRV)::ura4* | *clr3Δ::kanR ura4-DS/E leu1-32 ade6-210 his7-366 arg-* |
| **TP22** | *L(SacI)::ade6 K(XbaI)::ura4* | *clr3Δ::kanR ura4-D18 leu1-32 ade6-210 his2- arg-* |
| **TP38** | *mat2-M-SRE3 mat3-P-SRE2* | *ura4-D18 leu1-32 ade6-216* |
| **TP39** | *mat2-P-SRE3 mat3-M-SRE2* | *ura4-D18 leu1-32 ade6-216* |
| **TP48** | *mat2-PΔSRE2 mat3-MΔSRE3* | *ura4-DS/E leu1-32 ade6-216* |
| **TP75** | *mat3-MΔSRE3* | *ura4-D18 leu1-32 ade6-216* |
| **TP126** | *mat3-M-SRE2* | *ura4-DS/E leu1-32 ade6-216* |
| **TP133** | *h^90^* | *swi2Δ::kan ura4-D18 leu1-32 ade6-216* |
| **TP138** | *h^90^* | *swi5Δ::kan ura4-D18 leu1-32 ade6-216* |
| **TP149** | *mat2-PΔSRE2* | *swi5Δ::kan ura4-DS/E leu1-32 ade6-210* |
| **TP150** | *mat3-MΔSRE3* | *swi5Δ::kan ura4-DS/E leu1-32 ade6-210* |
| **TP153** | *mat2-PΔSRE2 mat3-MΔSRE3* | *swi5Δ::kan ura4-D18 leu1-32 ade6-216* |
| **TP156** | *mat2-PΔSRE2* | *swi2Δ::kan ura4-D18 leu1-32 ade6-210* |
| **TP157** | *mat3-MΔSRE3* | *swi2Δ::kan ura4-D18 leu1-32 ade6-216* |
| **TP160** | *mat2-PΔSRE2 mat3-MΔSRE3* | *swi2Δ::kan ura4-DS/E leu1-32 ade6-210* |
| **TP186** | *mat1-Msmt-0* | *swi2::13myc::kanR leu1-32* |
| **TP192** | *mat1-Msmt-0 mat3-MΔSRE3* | *swi2::13myc::kanR leu1-32* |
| **TP197** | *mat1-PD17::LEU2 mat3-MΔSRE3* | *swi2::13myc::kanR leu1-32* |
| **TP220** | *h^90^* | *ura4-D18 leu1::ura4-mfm3p+YFP-map2p+CFP ade6-216* |
| **TP221** | *h^90^* | *swi2Δ::kan swi6Δ::ura4 ura4-D18 leu1-32 ade6-210* |
| **TP262** | *mat2-M-SRE2 mat3-P-SRE3* | *ura4-D18 leu1::ura4-mfm3p+YFP-map2p+CFP ade6-216* |
| **TP263** | *mat2-M-SRE3 mat3-P-SRE2* | *ura4-D18 leu1::ura4-mfm3p+YFP-map2p+CFP ade6-216* |
| **TP265** | *mat2-P-SRE3 mat3-M-SRE2* | *ura4-D18 leu1::ura4-mfm3p+YFP-map2p+CF ade6-216* |
| **TP268** | *mat2-PΔSRE2* | *ura4-D18 leu1::ura4-mfm3p+YFP-map2p+CFP ade6-216* |
| **TP270** | *mat3-MΔSRE3* | *ura4-D18 leu1::ura4-mfm3p+YFP-map2p+CFP ade6-216* |
| **TP271** | *mat2-PΔSRE2 mat3-MΔSRE3* | *ura4-D18 leu1::ura4-mfm3p+YFP-map2p+CFP ade6-216* |
| **TP273** | *mat3-M-SRE2* | *ura4-D18 leu1::ura4-mfm3p+YFP-map2p+CFP ade6-216* |
| **TP291** | *mat2-M-SRE2 mat3-P-SRE3* | *swi6Δ::kan ura4-D18 leu1::ura4-mfm3p+YFP-map2p+CFP ade6-216* |
| **TP292** | *mat2-M-SRE3 mat3-P-SRE2* | *swi6Δ::kan ura4-D18, leu1::ura4-mfm3p+YFP-map2p+CFP ade6-216* |
| **TP293** | *mat3-MΔSRE3* | *swi6Δ::kan ura4-DS/E leu1::ura4-mfm3p+YFP-map2p+CFP ade6-216* |
| **TP294** | *mat2-P-SRE3 mat3-M-SRE2* | *swi6Δ::kan ura4-DS/E leu1::ura4-mfm3p+YFP-map2p+CFP ade6-216* |
| **TP295** | *mat2-PΔSRE2 mat3-MΔSRE3* | *swi6Δ::kan ura4-D18 leu1::ura4-mfm3p+YFP-map2p+CFP ade6-216* |
| **TP296** | *mat2-PΔSRE2* | *swi6Δ::kan ura4-D18 leu1::ura4-mfm3p+YFP-map2p+CFP ade6-210* |
| **TP299** | *h^90^* | *swi6Δ::kan ura4-DS/E leu1::ura4-mfm3p+YFP-map2p+CFP ade6-216* |
| **TP300** | *mat3-M-SRE2* | *swi6Δ::kan ura4-DS/E leu1::ura4-mfm3p+YFP-map2p+CFP ade6-216* |
| **TP303** | *mat2-P-SRE3 mat3-M-SRE3* | *ura4-D18 leu1-32 ade6-210* |
| **TP310** | *mat2-P-SRE3* | *swi6Δ::kan ura4-D18 leu1-32 ade6-210* |
| **TP313** | *mat2-P-SRE3 mat3-M-SRE3* | *ura4-D18 leu1::ura4-mfm3p+YFP-map2p+CFP ade6-210* |
| **TP332** | *mat2-PΔSRE2(460bp)* | *ura4-DS/E leu1-32 ade6-210* |
| **TP349** | *mat2-PΔSRE2(460bp)* | *swi6Δ::kanR ura4-DS/E leu1-32 ade6-210* |
| **TP350** | *mat2-PΔSRE2(460bp)* | *swi2Δ::kanR ura4-DS/E leu1-32 ade6-210* |
| **TP351** | *mat2-PΔSRE2(460bp)* | *swi5Δ::kanR ura4-DS/E leu1-32 ade6-210* |
| **TP366** | *mat1-PD17::LEU2 mat2-PΔSRE2* | *swi2::13myc::kanR leu1-32* |
| **TP367** | *mat1-Msmt-0 mat2-PΔSRE2* | *swi2::13myc::kanR leu1-32* |
