# Supplementary material for: Two Portable Recombination Enhancers Direct Donor Choice in Fission Yeast Heterochromatin
Source: PLoS Genet. 2013 Oct 24;9(10):e1003762. doi: 10.1371/journal.pgen.1003762 (PMC3812072; doi:10.1371/journal.pgen.1003762)
Supplement: Table S2 — Oligonucleotide sequences. (DOCX) [file pgen.1003762.s005.docx]

**Table S2.** **Oligonucleotide sequences.**

| **Name** | **Oligonucleotide sequence** |
| --- | --- |
| GTO-318 | 5` GACCATGGCTGCTATCCCCAAATTGGCTTACGC-3` |
| GTO-319 | 5`-GACCATGGCATATCTGTGTAATAGCTTGTTTGCC-3` |
| GTO-321 | 5`-GACTGCAGATCTTGAACACGAATTATGGACATCC-3` |
| GTO-324 | 5`-CCCATGGTATCCAAATATGTTTGTTTGGCCG-3` |
| GTO-325 | 5`-CACATGTGCGATGTATTATACTAGTTTGACG-3` |
| GTO-326 | 5`-CCCATGGTTTGTGATTATGCTGTTCAGCATTG-3’ |
| GTO-327 | 5`- CACATGTCCAATTTGGGGATAGCAGTACTTC-3` |
| TJO-10 | 5`-CCTTCAACTACTCTCTCTTC-3’ |
| TJO-11 | 5`-GCCTACTGTTAATATAATAAC-3’ |
| TJO-37 | 5`-CGCCCGGGATGGTGAGCAAGGGCGAGGAGC-3` |
| TJO-42 | 5`-CGCCTGCAGGTGCTACTTCGAGCACTGTAC-3` |
| TJO-43 | 5`-GCGTCGACTGTTAAGTAAAGAAAGTAAA-3` |
| TJO-44 | 5`-CGCTGCAGCGGAACTAGCACTACTATGG-3` |
| TJO-45 | 5`-GCGTCGACAATGTATGTGAAAAAAATAA-3` |
| 42-F | 5'-agcaatttagcactatattagccttt-3' |
| 42-R | 5'-ggagatgcgtatttgggaac-3' |
| 44-F | 5'-caacaacggattactaaaaacagc-3' |
| 44-R | 5'-tcttgccaattttgactcca-3' |
| 46-F | 5'-tgcttggtgtgatgatcattg-3' |
| 46-R | 5'-caataacttcgtggtattcgg-3' |
| SRE2∆-F | 5'-CAGATATTGGAAGACCGATGACTAC-3' |
| SRE2∆-R | 5'-AACCGTTAGGAAAATATACGATATTTCAGC-3' |
| 49-F | 5'-cattcttttgataagtcggtacttca-3' |
| 49-R | 5'-tggcgattcaacgattaaca-3' |
| 51-F | 5'-tcgagtttcgaaagtttttcc-3' |
| 51-R | 5'-catcgaaaatgaccaatcaca-3' |
| 62-F | 5'-aacgtatctacaaatttctcgg-3' |
| 62-R | 5'-cccatgtacgaagaatcagag-3' |
| 66-F | 5'-accacatatcaacggcacaa-3' |
| 66-R | 5'-tcaggagttgcttcatgttaagtt-3' |
| 6-F | 5'-tgcactcacaactacaatgga-3' |
| 6-R | 5'-ccagggtacattttctgatgttg-3' |
| 69-F | 5'-ttttactgccctgattctatcg-3' |
| 69-R | 5'-caaaatgtctatccgggtaagaag-3' |
| SRE3∆-F | 5'-ttttactgccctgattctatcg-3' |
| SRE3∆-R | 5'-GCGTTGAGATAACTTGCAAAG-3' |
| 71-F | 5'-ttttcacgtttgaagagcaa-3' |
| 71-R | 5'-ggaaatctcctgccgaagt-3' |
| *act1*-F | 5'-GAAGTACCCCATTGAGCACGG-3' |
| *act1*-R | 5'-CAATTTCACGTTCGGCGGTAG-3' |
