# Supplementary material for: Two Portable Recombination Enhancers Direct Donor Choice in Fission Yeast Heterochromatin
Source: PLoS Genet. 2013 Oct 24;9(10):e1003762. doi: 10.1371/journal.pgen.1003762 (PMC3812072; doi:10.1371/journal.pgen.1003762)
Supplement: Table S3 — Cell counts from fluorescence microscopy. (DOCX) [file pgen.1003762.s006.docx]

**Table S3. Cell counts from fluorescence microscopy.**

| **Strain** | **Number of P cells**  **(cyan)** | **Number of M cells**  **(yellow)** | **Number of mated cells** | **Total number of cells** |
| --- | --- | --- | --- | --- |
| **TP262** | 99 | 513 | 4 | 620 |
| **TP263** | 617 | 678 | 22 | 1339 |
| **TP313** | 250 | 732 | 15 | 1012 |
| **TP265** | 246 | 657 | 30 | 963 |
| **TP273** | 263 | 207 | 44 | 558 |
| **TP271** | 521 | 80 | 74 | 749 |
| **TP268** | 47 | 616 | 10 | 683 |
| **TP270** | 476 | 68 | 19 | 582 |
| **TP220** | 622 | 522 | 28 | 1200 |
