# Supplementary material for: Two Portable Recombination Enhancers Direct Donor Choice in Fission Yeast Heterochromatin
Source: PLoS Genet. 2013 Oct 24;9(10):e1003762. doi: 10.1371/journal.pgen.1003762 (PMC3812072; doi:10.1371/journal.pgen.1003762)
Supplement: Text S1 — Extended experimental procedures. (DOC) [file pgen.1003762.s007.doc]

**Text S1. Extended experimental procedures**

***S. pombe* strains.**

The genotypes of all strains used in this study are listed in Table S1.

**Construction of *SRE2* deletion strain.**

A clone of the 6.3 kb *mat2-P Hin*dIII fragment in pUC119 [1] was used to create nested deletions on the centromere-distal side of the *mat2-P* H1 homology box. The H1 homology box at *mat2-P* ends with an *Nco*I site. Two PCR products were generated by amplifying genomic DNA of the wild-type strain 968 [2] using GTO-318 (460 bp centromere-distal to H1, ending with added *Nco*I site) and the reverse primer GTO-321 (2291 bp centromere-distal to H1 ending with an added *Pst*I site) or GTO-319 (859 bp centromere-distal to H1, ending with added *Nco*I site) and GTO-321. The two PCR fragments digested with *Nco*I and *Pst*I were used to replace the H1-distal DNA in the *mat2-P* plasmid, resulting in H1-adjacent deletions of respectively 460 bp in the pT1 plasmid and 859 bp in the pT2 plasmid. Inserts were released from the plasmids bydigestion with *Sap*I and *Xma*I and used to transform the *S. pombe* strain TP1. TP1 contains two markers ectopically inserted near *mat2-P*, *L(SacI)::ade6+* and *K(XbaI)::ura4^+^* [3]. FOA-resistant Ade- transformants were selected and characterized by Southern blot to identify transformants with correct integrations.

**Construction of *SRE3* deletion strain.**

pGT70d424 [4], a plasmid containing the 4.2 kb *mat3-M Hin*dIII fragment with a 424 bp deletion centromere-distal to the *mat3-M* H1 homology box was digested with *Hin*dIII and used to transform the *S. pombe* strain PG3089. FOA-resistant transformants were selected and characterized by Southern blot to identify transformants in which the pGT70d424 insert had replaced *mat3-M(EcoRV)::ura4^+^*.

**Construction of a strain with a double deletion of SRE2 and SRE3.**

The *SRE2Δ* *SRE3Δ* mating-type region was constructed in two steps. First, the *ura4+* gene was inserted at the *Eco*RV site centromere-distal to *mat3-M* in the *SRE2Δ* strain TP2 using the *mat3-M:ura4* construct [5]. In a second step, the *ura4^+^* gene was replaced with the pGT70d424 insert [4] as above for the construction of *SRE3Δ*.

**Construction of *h^90^* with swapped elements, 2xSRE2, 2xSRE3 and *h^09^* with swapped elements.**

The strains were obtained by a combination of transformations and crosses.

A first set of plasmids were constructed to swap the SRE elements at *mat2-P* and *mat3-M*. The SRE2 and SRE3 elements were amplified by PCR using GTO-326 and GTO-327 (for SRE2), or GTO-324 and GTO-325 (for SRE3). The amplified fragments were cloned separately into the pCRII TOPO vector (Invitrogen) and later released with *Nco*I and *Pci*I for subsequent cloning into pT1 and pGT70d424. As described above pT1 contains the *mat2-P Hin*dIII fragment with an *Nco*I immediately adjacent to the SRE2 deletion at the edge of the H1 box while pGT70d424 contains the *mat3-M Hin*dIII fragment with an *Nco*I immediately adjacent to the SRE3 deletion. The SRE2-containing *Nco*I-*Pci*I fragment was cloned into pGT70d424 linearized with *Nco*I and a plasmid with SRE2 is in its native chromosomal orientation was identified, creating a 4.2 kb *mat3-M Hin*dIII clone where SRE3 is replaced with SRE2, pT7. Similarly, the SRE3-containing *Nco*I-*Pci*I fragment was cloned into pT1 linearized with *Nco*I and a plasmid in which SRE3 is in its native orientation was identified, creating a 6.3 kb *mat2-P Hin*dIII clone where SRE2 is replaced with SRE3, pT6. A second set of plasmids were constructed to swap the SRE elements in *mat2-M* and *mat3-P,* the *h^09^* cassettes. The 1.3kb *Nco*I-*Pst*I fragment of pGT65 (pGT65 contains *mat2-M*; [6]) was replaced with the *Nco*I-*Pst*I fragment of pT6 (containing SRE3 and *mat2*-distal DNA) to create the *mat2-MSRE3* cassette (pT4). The 0.9 kb *Nco*I-*Pst*I fragment of pGT66 (pGT66 contains *mat3-P*; [6]) was replaced with the *Nco*I-*Pst*I fragment of pT7 (containing SRE2 and *mat3*-distal DNA) to create the *mat3-PSRE2* cassette (pT5).

pT6 (*mat2-P-SRE3*) and pT4 (*mat2-M-SRE3*) were linearized with *Hind*III and used separately for integration in TP22, a *his2*^-^ strain with *L(SacI)::ade6^+^* and *K(XbaI)::ura4^+^* flanking the *mat2-P* cassette. pT7 (*mat3-M*-*SRE2*) and pT5 (*mat3-P-SRE2*) were linearized with *Hin*dIII and used separately for integration in TP20, a *his7*^-^ strain with *mat3-M(EcoRV)::ura4^+^*. The following strains were obtained from these transformations: *mat2-P*-SRE3 *mat3-M*-SRE3 *his2*^-^ (TP23); *mat2-M-SRE3 mat3-M-SRE3 his2*^-^ (TP24); *his7*^-^ *mat2-P-SRE2 mat3-M-SRE2* (TP25); *his7*^-^ *mat2-PSRE2 mat3-P-SRE2* (TP26). TP24 was crossed with TP26, His*^+^* recombinants were selected and progeny with a cross-over between *mat2* and *mat3* were identified by Southern blot, producing an *h^09^* strain with swapped elements, TP36. Similarly, TP23 was crossed with TP25, His^+^ recombinants were selected and progeny with a cross-over between *mat2* and *mat3* were identified by Southern blot, producing an *h^90^* strain with swapped elements, TP37.

All strain constructions were performed in a *clr3Δ* background to facilitate integration of constructs in the mating-type region and to permit cross-overs between *mat2* and *mat3*. *clr3Δ* was subsequently crossed out of strains of interest.

**Construction of cell-type specific fluorescent markers.**

To create a fluorescent reporter specifically expressed in M cells the *mfm3* promoter was amplified with TJO-42 and TJO-43 and cloned into pJET1.2 (Fermentas). The *mfm3* promoter was then released with *Sbf*I – *Sal*I and substituted for the *nmt*1 promoter in the RDB6118 50/E05 plasmid from RIKEN [7]. The resulting plasmid was amplified with TJO-43 and TJO-37 and religated to remove the *cig2* ORF. The final plasmid, pT22, encodes YFP under control of the *mfm3* promoter and can be integrated at the *leu1-32* locus to produce *leu1^+^* integrants. To create a fluorescent reporter specific for P cells, the *map2* promoter was first amplified with TJO-44 and TJO-45 and cloned into pJET1.2 (Fermentas). The pDUAL2-HFC1c [8] was linearized with *EcoR*V and religated to remove the ccdB(rfA) cassette. The *map2* promoter was released from pJET1.2 with *Pst*I-*Sal*I and used to replace the *nmt1* promoter in this modified pDUAL2-HFC1c. The final plasmid, pT27, encodes CFP under control of the *map2* promoter and can be integrated at the *leu1^+^* locus to produce *ura4^+^* *leu1^-^* integrants as described by [8].

The plasmid with the *mfm3*-YFP reporter (pT22 digested with *Not*I) was integrated first in the strains of interest followed by the plasmid with the *map2*-CFP reporter (pT27 digested with *Not*I).

**Sporulation phenotypes**

Sporulation efficiencies were assayed by staining colonies with iodine vapors as in [9].

**Southern blots**

*S. pombe* cells were propagated in liquid cultures (YES) overnight at 30ºC to saturation and genomic DNA for Southern blots was prepared as described in [10]. Genomic DNA was digested with *Dde*I to assay *mat1* content or with *Hind*III and *Hind*III + *BamH*I respectively to examine the structure of the mating-type region. The probe to analyze *mat1* content was a PCR product made with TJO-10 and TJO-11, corresponding to the centromere-proximal *Dde*I-*Nsi*I fragment. A mixture of 6.3 kb *mat2-P Hin*dIII fragment and 4.2 kb *mat3-M Hin*dIII fragment [11] was used to examine the overall structure of the *mat2-mat3* region. Strains with swapped elements were further tested by PCR and sequencing.

**Fluorescence microscopy**

Prior to microscopy cells were propagated in liquid medium (MSL low nitrogen with required supplements) overnight. An Imager.Z1 microscope from Zeiss equipped with a Orca-ER CCD camera (Hamamatsu, Japan) and an HXP 120C lamp from Leica, Germany was used for the fluorescence microscopy. Filter sets were from Chroma (Brattleboro, VT) band-pass CFP (31044v2) and YFP (41028). Images were acquired at 100-fold magnification with the Volocity software.

**Chromatin Immunoprecipitation (ChIP)**

ChIP was performed as in [12] starting from 250 ml cultures in EMM2 with required supplements. Strains with the *swi2^+^::13Myc::KAN* allele [13] were used for immunoprecipitation with c-*myc* Ab-2 antibody (Thermo Scientific). PCR was performed with Pfu or DreamTaq (Fermentas), using as primer pairs a subset of the primers in [14] also listed here in Table S2. The Pfu polymerase was used with primer pairs: 42, 44, 46, SRE2∆, 51, 66, 6; and DreamTaq was used with primer pairs: 49, 62, 69, SRE3∆, 71. The PCR products were separated on 1.5% agarose gels and visualized by ethidium bromide staining with a BioRad Gel imaging station. Band intensities were measured with the ImageQuant software (GE Healthcare Life Sciences). Fold enrichments were calculated according to the formula: Fold enrichment = (*mat*_IP_/*act1*_IP_) / (*mat*_WCE_/*act1*_WCE_).

**References**

1. Kelly M, Burke J, Smith M, Klar A, Beach D (1988). Four mating-type genes control sexual differentiation in the fission yeast. EMBO J 7: 1537-1547.
2. Leupold U (1950) Die Vererbung von Homothallie und Heterothallie bei *Schizosaccharomyces pombe*. Compt Rend Trav Lab Carlsberg SQr Physiol 24: 381-480.
3. Ayoub N, Goldshmidt I, Cohen A (1999) Position effect variegation at the mating-type locus of fission yeast: a cis-acting element inhibits covariegated expression of genes in the silent and expressed domains. Genetics 152: 495-508.
4. Thon G, Bjerling KP, Nielsen IS (1999) Localization and properties of a silencing element near the mat3-M mating-type cassette of Schizosaccharomyces pombe. Genetics 151: 945-963.
5. Thon G, Klar AJ (1992) The clr1 locus regulates the expression of the cryptic mating-type loci of fission yeast. Genetics 131: 287-96.
6. Thon G, Klar AJ (1993) Directionality of fission yeast mating-type interconversion is controlled by the location of the donor loci. Genetics 134: 1045-1054.
7. Matsuyama A, Arai R, Yashiroda Y, Shirai A, Kamata A, et al. (2006) ORFeome cloning and global analysis of protein localization in the fission yeast Schizosaccharomyces pombe. Nature Biotechnol 24: 841-847.
8. Matsuyama A, Shirai A, Yoshida M (2008) A novel series of vectors for chromosomal integration in fission yeast. Biochem Biophys Res Commun 374: 315-319.
9. Bresch C, Muller G, Egel R (1968) Genes involved in meiosis and sporulation of a yeast. Mol Gen Genet 102: 301-306.
10. Moreno S, Klar A, Nurse P (1991) Molecular genetic analysis of fission yeast Schizosaccharomyces pombe. Methods Enzymol 194: 795-823.
11. Beach DH (1983) Cell type switching by DNA transposition in fission yeast. Nature 305: 682-687.
12. [Volpe TA](http://www.ncbi.nlm.nih.gov/pubmed?term=Volpe TA%5BAuthor%5D&cauthor=true&cauthor_uid=21528444), [Demaio J](http://www.ncbi.nlm.nih.gov/pubmed?term=Demaio J%5BAuthor%5D&cauthor=true&cauthor_uid=21528444) (2011) Chromatin immunoprecipitation in fission yeast. Methods Mol Biol 725:15-28.
13. Aguilar-Arnal L, Marsellach FX, Azorín F (2008) The fission yeast homologue of CENP-B, Abp1, regulates directionality of mating-type switching. EMBO J 27: 1029-1038.
14. Matsuda E, Sugioka-Sugiyama R, Mizuguchi T, Mehta S, Cui B, et al. (2011) A homolog of male sex-determining factor SRY cooperates with a transposon-derived CENP-B protein to control sex-specific directed recombination. Proc Natl Acad Sci USA 108: 18754-18759.
